# Scaffold Sheet Design Strategy for Soft Tissue Engineering †

**DOI:** 10.3390/ma3021375

**Published:** 2010-02-24

**Authors:** Richard T. Tran, Paul Thevenot, Yi Zhang, Dipendra Gyawali, Liping Tang, Jian Yang

**Affiliations:** Department of Bioengineering, University of Texas at Arlington, 501 West First Street, Arlington, TX 76019, USA; E-Mails: mrtran@uta.edu (R.T.); pthevenot@tx.rr.com (P.T.); zhangyi19850520@gmail.com (Y.Z.); dipendra1411@hotmail.com (D.G.); ltang@uta.edu (L.T)

**Keywords:** biodegradable, elastomer, 3D tissue construction, scaffold sheet, *in vivo* tissue engineering

## Abstract

Creating heterogeneous tissue constructs with an even cell distribution and robust mechanical strength remain important challenges to the success of *in vivo* tissue engineering. To address these issues, we are developing a scaffold sheet tissue engineering strategy consisting of thin (~200 μm), strong, elastic, and porous crosslinked urethane-doped polyester (CUPE) scaffold sheets that are bonded together chemically or through cell culture. Suture retention of the tissue constructs (four sheets) fabricated by the scaffold sheet tissue engineering strategy is close to the surgical requirement (1.8 N) rendering their potential for immediate implantation without a need for long cell culture times. Cell culture results using 3T3 fibroblasts show that the scaffold sheets are bonded into a tissue construct via the extracellular matrix produced by the cells after 2 weeks of *in vitro* cell culture.

## 1. Introduction

Tissue engineering is a multi-disciplinary field that combines the principles of polymer chemistry, engineering, and biological sciences in efforts to develop biological substitutes to improve or replace the functions of failing tissues and organs [1]. One of the main principle methods behind tissue engineering involves growing relevant cells into a three-dimensional (3D) tissue or organ. Although cells alone lack the ability to grow into 3D orientations similar to the native tissues, the preferred multidimensional cellular growth is achieved by seeding the desired cells onto porous matrices, known as scaffolds [2]. By serving as a temporary suitable microenvironment for extracellular matrix (ECM) and 3D tissue formation, the scaffold has become a very important component of tissue engineering [3].

Scaffold requirements for tissue engineering are multifaceted and particular to the structure and function of the tissue of interest [4]. Many scaffold processing and fabrication techniques such as fiber meshes [5], phase separation [6], solvent casting and particulate leaching [7], membrane lamination [8], and melt molding [9] have been utilized in a wide variety of applications including bone [10], cartilage [11], blood vessels [12], and heart valves [13]. Although these scaffolds have demonstrated promise, uneven cell distribution and nutrient delivery in the deep portion of the synthetic scaffolds (> 200 μm) due to the random mobility of cell suspension often compromise their successful uses in tissue engineering [14,15]. The seeded cells and matrix produced by cells at the scaffold periphery also act as a barrier to the diffusion of oxygen and nutrients into the interior of the scaffold. Unfortunately, these constraints limit the majority of successful tissue engineering applications to the maximum diffusion distance of 200 μm [16,17].

A pioneering 2D cell-sheet tissue engineering strategy was proposed to regenerate several types of tissues such as blood vessel [18], skin [19], corneal epithelium [20], urothelium [21], and periodontal ligament [22]. By using a layer-by-layer technique, stratified tissues were created from the stacking of single cell sheets to create more complex structures such as liver lobules and kidney glomeruli [23]. Although problem of uneven cell distribution is not a concern in this case, cell sheets are too fragile to handle and the cell-sheet constructs require long *in vitro* culture time to mature prior to implantation, which is a considerable limitation of this technology [24]. Also, cells grown in a 2D culture dishes could bring some concerns such as losing important cell characteristics during the long-term culture, and only a limited number of cell types are able to form cell sheets thereby restricting the potential to engineer various types of tissues [18].

In this study, we show the proof of concept for the scaffold sheet tissue engineering strategy using thin and elastic crosslinked urethane-doped polyester (CUPE) scaffolds. CUPEs are a new class of biodegradable elastomers that we have been developing to engineer soft, but strong scaffolds for soft tissue engineering applications. CUPEs are easy to synthesize, potentially cost effective, and have demonstrated a wide range of mechanical properties with tunable degradation profiles [25]. In this work, we introduce a new scaffold sheet design with the following advantages: (1) Single CUPE scaffold sheets can be fabricated and post-polymerized together to form complex constructs composed of layers with different pore structures. (2) Single CUPE scaffold sheets can be seeded with cells and stacked together similar to the 2D cell-sheet tissue engineering strategy to form stratified tissues. (3) The use of thin (~200 μm thick) scaffold sheets should address the issues of uneven cell distribution, which is a common concern in the use of synthetic scaffolds for tissue engineering applications. (4) The CUPE scaffolds can provide a 3D microenvironment for cell proliferation without the potential loss of cell characteristics. (5) The scaffold sheet design strategy in combination with a layer-by-layer approach has the potential to enable the compartmentalization of multiple cell types in the engineering of complex tissues. (6) The scaffold sheets are strong and compliant so that they may be implanted *in vivo* soon after cell seeding, which should eliminate the long *in vitro* culture times needed for construct maturation. The CUPE scaffold sheets were evaluated via mechanical tests and cell culture experiments to assess its potential use in soft tissue engineering.

## 2. Results and Discussion

Many scaffold fabrication techniques previously reported have resulted in an uneven distribution of cells within the construct [13,14,15]. In the case of most *in vivo tissue*, a network of vascularization offers a maximum nutrient diffusion distance of about 200 μm, which is most likely the reason why most successful tissue engineered applications have resulted in the growth of tissues with cross sections less than 500 μm from the external surface of the scaffold [17,26]. In this study, we report on the feasibility of using a new scaffolding design strategy named scaffold sheet tissue engineering. The concept behind this new approach involves the use of thin (~200 μm) strong and compliant scaffold sheets in combination with a layer-by-layer approach to form stratified 3D constructs without the issues of uneven cell distribution or the potential loss of cell characteristics seen in 2D cell-sheet tissue engineering. 

**Figure 1:**
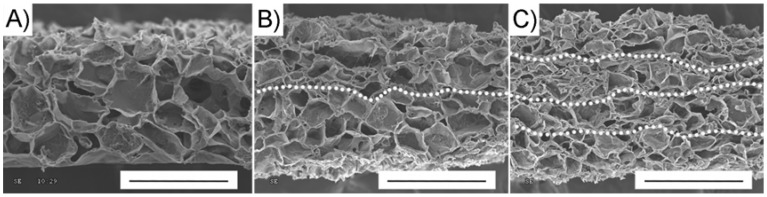
Representative SEM image cross-sections (a) single CUPE 1.2 scaffold sheet (scale bar = 150 μm), (b) two CUPE 1.2 scaffold sheets crosslinked together (scale bar = 300 μm), and **(c)** 4 CUPE 1.2 scaffold sheets crosslinked together (scale bar = 600 μm).

**Scheme 1:**
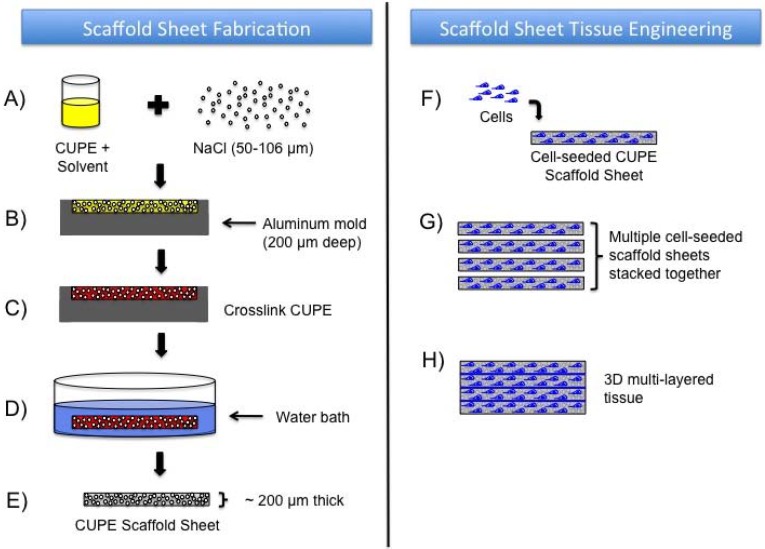
A schematic representation of the scaffold sheet fabrication process and scaffold sheet tissue engineering design. (a) Pre-crosslinked urethane-doped polyester (pre-CUPE) is mixed with sieved salt (50–106 μm). (b) The resulting polymer/salt slurry is cast into an aluminum mold (~200 μm deep). (c) The polymer filled aluminum mold is placed in an oven for predetermined times for post-polymerization to crosslink the polymer. (d) After post-polymerization, the salt is leached from the scaffold by immersion in water. (e) Next, the scaffold is lyophilized to remove any trace amounts of water. The result is a thin (~200 μm thick), soft, and elastic CUPE scaffold sheet. (f) The sterilized CUPE scaffold sheets are seeded with cells. (g) Multiple cell-seeded CUPE scaffold sheets are stacked together and allowed to culture. (h) The bonded scaffold sheets produce a three-dimensional multi-layered construct through a layer-by-layer technique.

The morphology of the CUPE scaffold sheets were fabricated according to Scheme 1A-E, and were verified using SEM and NIH ImageJ analysis software. Figure 1 presents SEM images of a single porous CUPE scaffold sheet and multiple scaffold sheets stacked and crosslinked (or post-polymerized) together. The scaffold sheets produced from this technique displayed an overall scaffold thickness of 179.16 ± 8.16 μm, porosity of 86.60 ± 3.05%, and interconnected pore size 78.04 ± 13.59 μm (Figure 1A). The pore sizes created from this technique were limited to less than 150 μm due to restrictions in the depth of the machined aluminum mold. The above parameters, however, can easily be adjusted by changing the mold dimensions, polymer: salt ratio, and porogen size to fit the needs of a specific application. The images shown in Figure 1B and C represent the cross-sections of two and four scaffold sheets crosslinked together, respectively, showing the ability to stack multiple layers together to form one continuous construct. This layer-by-layer technique opens a way to fabricate complex heterogeneous porous scaffolds with different porosities, pore sizes, pore shape, and interconnectivity amongst each layer while maintaining a porous interface to accommodate for specific cell types in the regeneration of multi-cellular tissues such as skin, blood vessels, liver, pancreas, and cartilage where the compartmentalization of different cell types is necessary [27,28,29].

Early efforts in tissue engineering have mainly focused on the use of biodegradable synthetic polymers, such as polylactic acid (PLA), polyglycolic acid (PGA), polycaprolactone (PCL), and their copolymers, to support ECM production. Unfortunately, these previous materials have been limited in their success due to their lack of strength and elasticity causing a mismatch in compliance [30,31,32,33,34,35]. Biodegradable elastomers, such as poly (octamethylene citrates) (POC), poly (glycerol sebacate) (PGS), and more recently crosslinked biodegradable photoluminescent polymer (CBPLP) have received much attention recently as materials for vascular tissue engineering due to their elastic nature and excellent cell/tissue compatibility [36,37,38,39,40,41,42,43,44,45]. However, these materials lack the sufficient strength when fabricated into porous scaffolds. In an effort to add strength while maintaining elasticity, we have recently reported on the synthesis and characterization of a novel biomaterial, CUPE, which combines the elasticity of polyesters with the strength of polyurethanes. A simple doping of a urethane group into the POC network resulted in films with a 10-fold increase in tensile strength, while maintaining a soft and elastic nature [25].

**Figure 2:**
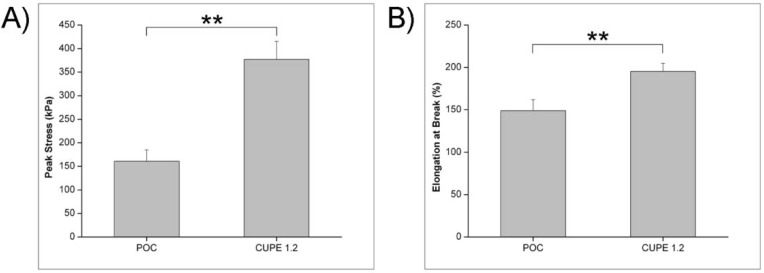
A comparison between single POC and CUPE 1.2 scaffold sheets. All scaffolds were post-polymerized at 80 °C for 4 days followed by 120 °C for 1 day under vacuum. (a) Effect of scaffold material on the peak stress. (b) Effect of scaffold material on elongation at break. ** p < 0.01; N = 6.

A comparison between the mechanical properties of POC and CUPE scaffold sheets are shown in Figure 2. A significant increase in the peak tensile stress (160.67 ± 24.48 kPa to 377.12 ± 36.87 kPa) and elongation at break (149.09 ± 12.78% to 195.29 ± 11.80%) was observed when CUPE was used as the material for scaffold sheet fabrication. The enhanced mechanical properties from the addition of HDI into the POC pre-polymer can be explained due to the increased hydrogen bonding between the urethane groups, which are not present in the POC scaffolds, to produce higher peak tensile strength while preserving the elasticity of the material.

**Figure 3:**
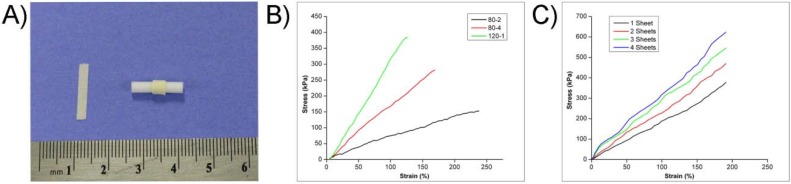
(a) Photograph of a single CUPE 1.2 scaffold sheet (left) and CUPE 1.2 scaffold sheet rolled onto a Teflon rod (right). Typical tensile stress-strain curves of (b) single CUPE 1.2 scaffold sheets post-polymerized under different reaction conditions and (c) multiple CUPE 1.2 scaffold sheets post-polymerized at 80 °C for 4 days followed by 120 °C for 1 day under vacuum.

Figure 3A presents a photomicrograph of a single CUPE 1.2 scaffold sheet, and a scaffold sheet rolled around a Teflon rod. All CUPE scaffold sheets fabricated in this study were soft, elastic, and showed 100% recovery after deformations with the ability to be folded and rolled into any desired shape without kinking. Based on the stress-strains of the scaffold sheets, the tensile strength, initial modulus, and elongation at break were calculated. As shown in Figure 3A, the stress-strains curves were characteristic of elastomers with no yield point in any of the scaffold mechanical tests. Multiple scaffold sheet stress-strain curves produced single breaks indicating that the individual sheets were bonded together through the post-polymerization process (Figure 3C).

The compliance mismatch between the scaffold and host tissue is a biomechanical problem that has been cited to inhibit the successful integration of the construct with native tissue, and ultimately results in implant failure [27]. Thus, the ability to engineer the appropriate scaffold mechanical properties to suit a particular application plays an important role in the success of the implant. One advantage in the CUPE scaffold sheet design is that the mechanical properties can be controlled through a variety of options such as the post-polymerization condition, pre-polymer chemistry, and the number of scaffold layers. The mechanical properties of single CUPE 1.2 scaffold sheets post-polymerized under different reaction conditions are summarized in Table 1. As the post-polymerization time and temperature were increased, a significant increase in the peak stress, initial modulus, and suture retention strength was observed with a corresponding drop in elasticity. A significant increase in the peak stress was observed from 2 days at 80 °C to 4 days at 80 °C (150.25 ± 17.38 to 294.54 ± 38.30 kPa, respectively). The same trend was also displayed as the post-polymerization temperature was increased from 80 °C to 120 °C.

**Table 1:** Mechanical properties of single CUPE 1.2 scaffold sheets (thickness 179.16 ± 8.16 μm; porosity 86.60 ± 3.05%; pore size 78.04 ± 13.59 μm) post-polymerized under different reaction conditions.^a^

Post-polymerization	Peak Stress	Initial Modulus	Elongation at Break	Suture Retention
Condition	(kPa)	(MPa)	(%)	(N)
80°C-2	150.25 ± 17.38	0.07 ± 0.01	230.63 ± 9.25	0.07 ± 0.01
80°C-4	294.54 ± 38.30	0.15 ± 0.02	212.08 ± 9.68	0.10 ± 0.01
120°C-1	377.12 ± 36.87	0.21 ± 0.02	195.29 ± 11.80	0.12 ± 0.01

^a^ Values are given as the means standard deviation. N = 6.

Although the elongation at break was sacrificed with the harsher post-polymerization conditions, single CUPE 120 °C-1 scaffold sheets showed a maximum peak stress of 377.12 ± 36.87 kPa with an elongation at break of 195.29 ± 11.80%, which is close to the elasticity and elongation of native vessels [46]. In the case of *in vivo* tissue engineering, the elastomeric nature of the CUPE scaffolds can permit the transmission of dynamic mechanical stimuli exerted by the cardiovascular system. The increase in mechanical properties from the harsher post-polymerization conditions was contributed to an increase in the crosslinking density of the resulting material to create a stronger and stiffer scaffold. By varying the post-polymerization conditions, the mechanical properties of the scaffold sheets can be tuned for the intended application.

**Figure 4:**
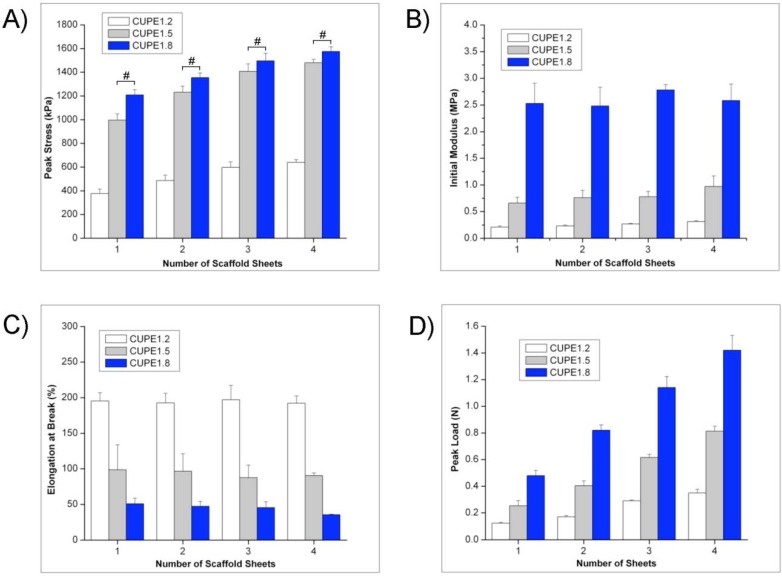
(a) Tensile peak stress, (b) initial modulus, (c) elongation at break, and (d) suture retention strength of multiple scaffold sheets fabricated using different CUPE HDI ratios. All scaffolds were post-polymerized at 80 °C for 4 days followed by 120 °C for 1 day under vacuum.

The effects of the pre-polymer isocyanate concentration on the resulting construct mechanical strength were also evaluated (Figure 4). A significant increase in the scaffold peak stress and initial modulus with a corresponding decrease in elasticity was observed as the pre-polymer HDI concentration was increased. However, no difference was observed in peak stress for CUPE 1.5 and CUPE 1.8 scaffold sheets (p > 0.05). In addition to the pre-polymer compositions, the effects of the number of stacked scaffold sheet layers on the mechanical properties were determined. As the number of stacked scaffold sheet layers was increased, an increasing trend in the peak stress was observed. However, the results also showed that the number of stacked scaffold sheets had no effect on the construct initial modulus or elongation at break (Figure 4B and C). By increasing the number of scaffold layers, the CUPE scaffold sheet design strategy can potentially meet the mechanical surgical requirements of implantation.

The suture retention strength is a crucial factor in the fabrication of tissue engineering scaffolds as it directly relates to the success of the construct during the implantation process. In these experiments, both an increase in the pre-polymer HDI concentration and number of scaffold sheets resulted in higher suture retention strengths (Figure 4D). Although, the suture retention strength for single CUPE 1.2 scaffold sheets were relatively low (0.12 ± 0.01 N), CUPE 1.8 scaffold sheets, when stacked into 4 layers, were close to the accepted adequate suture retention strength of 1.8 N [47].

**Figure 5:**
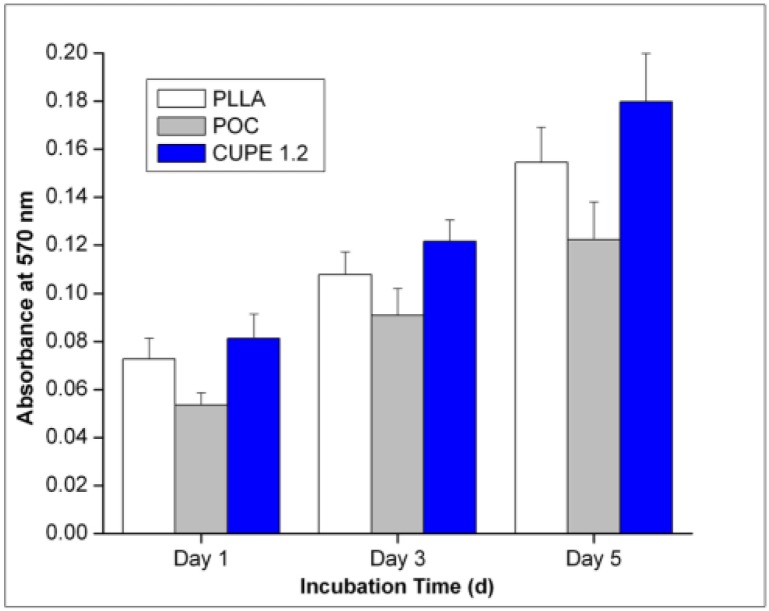
A comparison of 3T3 fibroblast growth and proliferation on single PLLA, POC, and CUPE 1.2 scaffold sheets over a 5-day incubation time period. MTT absorption was measured at 570 nm. N = 6.

3T3 cell adhesion and proliferation were quantitatively assessed using the MTT assay. Results indicated that a larger number of cells attached and proliferated to the CUPE scaffolds when compared to that of POC scaffolds at all time points. However, no significant difference in cell growth was observed when comparing the CUPE scaffolds to that of PLLA control scaffolds (Figure 5). Future studies will involve the *in vitro* cell compatibility using other cell lines such as human aortic endothelial and smooth muscle cells to evaluate the cell compatibility of the CUPE scaffold sheets for multi-cellular applications. 

**Figure 6:**
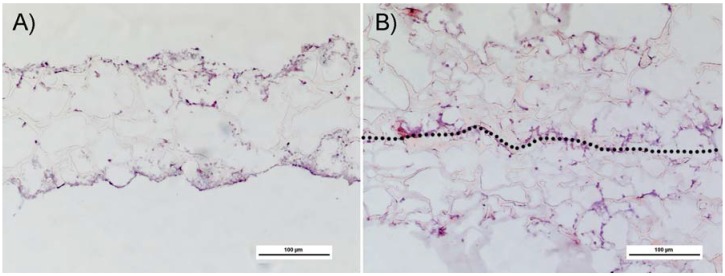
Photomicrographs of H&E stained CUPE 1.2 cross-sections after 2 weeks of cell culture. (A) Single scaffold sheet. (B) 2 scaffold sheets were shown to have bonded together using 3T3 fibroblasts (scale bar = 100 μm).

Although we understand that chemically bonded scaffold sheet constructs may not relate to cell culture bonded constructs, a quick screen to fabricate tissue interlocked constructs was performed to assess the feasibility of the approach. The layer-by-layer fabrication process depicted in Scheme 1F-H was evaluated using 3T3 fibroblast cell culture to bond individual scaffold sheets. H&E stained cross-sections of cell seeded scaffolds show that cells attached and proliferated along the exterior with an even distribution throughout the interior of the scaffold (Figure6A). The photomicrographs shown in Figure 6B present the H&E stained cross-sections of two scaffold sheets stacked together and allowed to culture over a two-week time period. The scaffold layers were shown to have bonded between the interfaces of each scaffold layer through ECM production by the seeded cells. These results provide evidence that the scaffold sheet design can potentially be used to combine multiple seeded scaffold sheets to produce a stratified construct.

## 3. Experimental Section

All chemicals, cell culture medium, and supplements were purchased from Sigma-Aldrich (St. Louis, MO, USA), unless mentioned otherwise, and used as received.

### 3.1. Crosslinked urethane-doped polyester (CUPE) pre-polymer synthesis

CUPE pre-polymers were synthesized in two distinct steps similar to previously published methods [25]. The first step involves the synthesis of a POC soft segment, which is chain extended by 1,6-hexamethyl diisocyanate (HDI) in the second polyurethane synthesis step. Briefly, a POC pre-polymer was first synthesized by reacting a 1:1.1 monomer ratio of citric acid and 1,8-octanediol, respectively, in a three-necked round bottom flask fitted with an inlet and outlet adapter at 160 °C under a constant flow of nitrogen [36]. Once all the monomers had melted, the temperature of the system was lowered to 140 °C, and the reaction mixture was allowed to continue for 60 minutes to create the POC pre-polymer. The POC pre-polymer was then purified by drop-wise precipitation in deionized water. The undissolved pre-polymer was collected and lyophilized for 48 hours to obtain the pre-POC soft segment.

In the second step, chain extension was achieved by dissolving pre-POC in 1,4-dioxane (3 wt %), and the resulting solution was allowed to react with HDI in a clean reaction flask under constant stirring at 55 °C using stannous octoate as a catalyst (0.1 wt %). Various CUPE pre-polymers were synthesized using three different molar feeding ratios of the pre-POC:HDI (1:1.2, 1:1.5, and 1:1.8 which are referred to as pre-CUPE 1.2, pre-CUPE 1.5, and pre-CUPE 1.8, respectively). The reaction was terminated upon the disappearance of the isocyanate peak located at 2267 cm^-1^, which was determined by FT-IR analysis.

### 3.2. CUPE scaffold sheet fabrication

CUPE scaffold sheets were fabricated using a particulate leaching technique and according to the procedures illustrated in Scheme 1. Briefly, a pre-CUPE solution was mixed with sieved sodium chloride salt (99% purity) with an average pore size in the range of 50–106 μm in a (1:9) polymer to salt ratio by weight (Scheme 1A). The polymer solution was mixed thoroughly with the salt until a viscous paste was formed. The resulting slurry was cast onto an aluminum mold machined to have a cavity approximately 200 μm deep (Scheme 1B), and then placed in a laminar flow hood overnight for solvent evaporation. Once the solvent was removed, the scaffold sheets were post-polymerized in an oven maintained at 80°C (Scheme 1C) for predetermined times to crosslink the pre-CUPE into CUPE. Next, the salt in the scaffold was leached out by immersion in deionized water for 72 hours with water changes every 12 hours (Scheme 1D). Finally, the scaffolds were lyophilized for 36 hours to remove any residual water.

POC and PLLA scaffold sheets were also fabricated as described above to serve as a control. To study the effects of the post-polymerization conditions, single scaffold sheets were placed in an oven maintained at 80 °C for 2 days (80 °C-2), 80 °C for 4 days (80 °C-4), and 80 °C for 4 days followed by additional post-polymerization at 120°C under vacuum for 1 day (120 °C-1). To study the effects of the number of scaffold layers, multiple pre-CUPE scaffold sheets were stacked together and post-polymerized at 120 °C-1. To study the effects of the HDI ratio on the resulting constructs, pre-CUPE 1.2, pre-CUPE 1.5, and pre-CUPE 1.8 were used to fabricate scaffold sheets and post-polymerized at 120 °C-1.

### 3.3. CUPE scaffold sheet characterization

To view the cross-sectional morphology, CUPE scaffold sheet samples were freeze-fractured using liquid nitrogen, sputter coated with silver, and examined under a Hitachi S-3000N scanning electron microscope (SEM) (Hitachi, Pleasanton, CA, USA). Image J analysis software was used to determine the scaffold sheet thickness. To characterize the scaffold geometries, 3 random locations were selected and a total of 30 measurements were recorded. The dimensions reported are expressed as the means ± standard deviation.

The scaffold porosity was measured using the Archimedes’ Principle similar to previously published methods [48]. Briefly, a density bottle was used to measure the density and porosity of the scaffold using ethanol (density ρ_e_) as the displacement liquid at 30°C. The density bottle filled with ethanol was weighed (W_1_). A scaffold sample of weight W_S_ was immersed into the density bottle, and the air trapped in the scaffold was evacuated under vacuum. Next, the density bottle was supplemented with ethanol, filled, and weighed (W_2_). The ethanol-saturated scaffold was removed from the density bottle, and the density bottle was weighed (W_3_). The following parameters of the scaffold were calculated: the volume of the scaffold pore (V_P_), the volume of the scaffold skeleton (V_S_), the density (ρ_S_), and the porosity (ε). The following formulas for the volume-mass index (V_P_/V_S_) were used [48]:

V_P_ = (W_2_ - W_3_ - W_S_) / ρ_e_
V_S_ = (W_1_ - W_2_ + W_S_) / ρ_e_
ρ_S_ = W_S_ / V_S_ = W_S_ρ_e_ / (W_1_ - W_2_ + W_S_)
ε = V_P_ / (V_P_ + V_S_) = (W_2_ - W_3_ - W_S_) / (W_1_ - W_3_)

The porosity of single scaffold sheets was recorded, and the results are reported as the means ± standard deviation (N = 6).

Tensile mechanical testing was conducted according to ASTM D412A standard on a MTS Insight 2 fitted with a 10 N load cell (MTS, Eden Prairie, MN, USA). Briefly, scaffold sheet strips (10 mm length × 2 mm width) were pulled at a rate of 500 mm/minute and elongated to failure. Values were converted to stress-strain and the initial modulus was calculated from the initial gradient of the resulting curve (0–10% elongation). The results are presented as the means ± standard deviation (N = 6). The suture retention strength was obtained similar to previously reported methods [49]. Briefly, one end of a CUPE scaffold sheet sample (10 mm length × 5 mm width) was fixed with the stage clamp of the tester, and the other end was connected to the opposite clamp through the suture material (5–0 Prolene, Ethicon, Piscataway, NJ, USA). The suture was placed 2 mm from the end of the sample. The measurement was performed using a 10 N load cell, and pulled at a rate of 8 mm/min until failure. The load at rupture was recorded (N), and the results are reported as the means ± standard deviation (N = 6).

### 3.4. In vitro cell culture attachment and proliferation

Cell compatibility of the CUPE scaffold sheets was evaluated *in vitro* using both quantitative and qualitative methods using NIH 3T3 fibroblasts (ATCC) as model cells. A quantitative assessment of the cell proliferation was performed using a Methylthiazoletetrazolium (MTT) cell proliferation and viability assay kit. CUPE 1.2, PLLA, and POC scaffold sheet samples were cut into cylindrical discs (7 mm in diameter) and sterilized in 70% ethanol for 3 hours. After incubation in ethanol, the samples were exposed to UV light for 30 minutes, and washed with phosphate buffered saline (PBS). The cells were cultured in Dulbecco’s modified eagle’s medium (DMEM), which had been supplemented with 10% fetal bovine serum (FBS) and 1% penicillin streptomycin. The culture flasks were kept in an incubator maintained at 37 °C, 5% CO_2_, and 95% relative humidity. The cells were allowed to grow to the fourth passage, trypsinized, centrifuged, and suspended into media to obtain a seeding density of 1 × 10^5^ cells/mL. MTT Assay analysis was performed at 1, 3, and 5 days of culture. At the pre-determined time point, the assay was conducted as per the manufacturer’s protocol. Briefly, the old media was aspirated, and each sample was washed with PBS to remove any loosely attached or dead cells. Next, 100 μL of 3-(4,5-dimethylthiazol-2yl)-diphenyltetrazolium bromide solution was then added to the samples, and allowed to incubate for 3 hours. At the end of the incubation period, the mixture of the MTT solution and incomplete media was aspirated and replaced with 100 μL of MTT solvent. Dissolution of the formazan crystals was facilitated by constant agitation of the well plate on an orbital shaker for 20 minutes. The absorbance was measured with an Infinite 200 microplate reader (Teacan Group Ltd., Switzerland) at 570 nm, with a reference wavelength of 690 nm, within 30 minutes of MTT solvent addition.

To feasibility of the scaffold sheet tissue engineering design strategy was evaluated according to the procedure described in Scheme 1. CUPE 1.2 scaffold sheet strips were prepared for cell culture as described above. Cells were seeded on both sides of the scaffold sheets at a density of 1 × 10^6^ cells/mL (Scheme 1F). After 1 day of culture, the scaffold sheets are stacked together (Scheme 1G), and allowed to culture for a two-week time period with media change every third day. After 2 weeks, the cells were fixed with the addition of cold methanol for 10 minutes and dried under vacuum. Scaffolds were then embedded in a liquid gelatin-sucrose solution, placed under vacuum for 30 minutes, and frozen at -20°C. Cross-sections of the scaffold were cut at 10 μm and hematoxylin and eosin (H&E) stained to visualize cell penetration, growth throughout the scaffold, and scaffold sheet bonding (Scheme 1H). 

## 4. Conclusions

We have explored the feasibility of a new tissue engineering strategy, named scaffold sheet tissue engineering. Porous, strong, and thin CUPE scaffold sheets were prepared using a particulate leaching technique to fabricate complex 3D scaffolds and bonded together either chemically or through cell culture using a layer-by-layer approach. In the chemically bonded procedure, single scaffold sheets were crosslinked together to show the potential in creating complex 3D constructs fabricated from individual layers composed of different morphologies. Unlike cell-sheet tissue engineering, the scaffold sheet tissue engineering cell bonded design implies that cells will be seeded on a thin scaffold sheet, which may provide a suitable environment similar to the natural extracellular matrix, and subsequently bonded together to potentially form multi-cellular stratified tissues. When stacked together, CUPE scaffold sheets displayed mechanical properties adequate for immediate implantation in potential applications for *in vivo* tissue engineering. Cell culture experiments show the cytocompatibility of the scaffold sheets, and the potential of the individual layers to be bonded together using the deposited cellular ECM. Future work will focus on using the scaffold sheet design to compartmentalize different cell types on various scaffold layers in the construction of multi-layer tissues (e.g. blood vessels), and to study the cell-cell communication between scaffold layers.
